# P-1925. Incidence of long COVID symptoms during the year post admission among patients hospitalized for COVID-19

**DOI:** 10.1093/ofid/ofae631.2085

**Published:** 2025-01-29

**Authors:** Mark Berry, Valentina Shvachko, Amanda Kong, Rohan Shah, Gina Brown, Anand Chokkalingam

**Affiliations:** Gilead Sciences, Inc., Foster City, California; Gilead Sciences, Inc., Foster City, California; Aetion, New York, New York; Aetion, Inc, New York, New York; Gilead Sciences, Foster City, California; Gilead, Foster City, California

## Abstract

**Background:**

The CDC defines long COVID as new, returning or ongoing health problems occurring at least 4 weeks after infection. However, it is uncertain how long different conditions may last after the infection. Our objective was to characterize incidence of common conditions that may be associated with long COVID across the first year after admission among patients hospitalized for COVID-19.

**Table 1.**
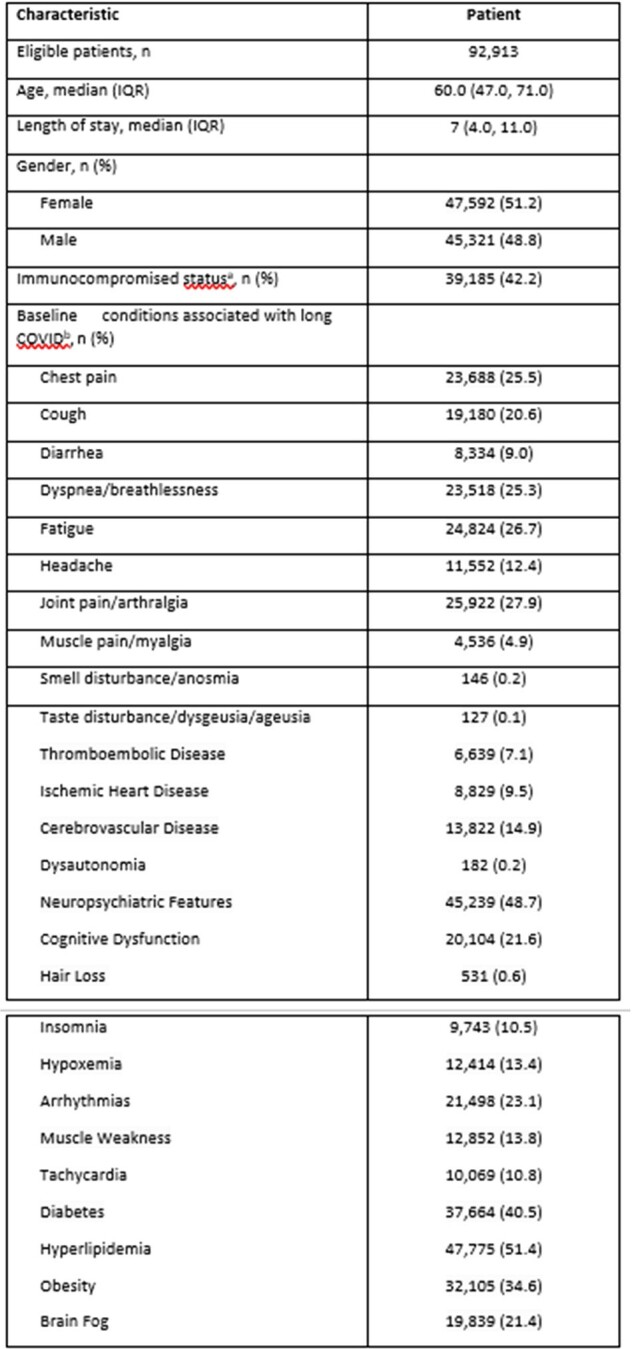
Patient Demographics IQR, interquartile range a Immunocompromised condition during the past 12 months (symptomatic HIV infection, haematologic and solid malignancy, organ transplant, rheumatologic/inflammatory, or other immune conditions).1. Patel M, et al. Emerg Infect Dis. 2020;26(8):1720-1730 b Measured 365 to 15 days prior to admission

**Methods:**

We identified patients hospitalized with COVID-19 from May 1, 2020-January 21, 2024 and with at least 31 days of followup in the HealthVerity claims and hospital chargemaster database. Patients with a diagnosis of long COVID (ICD-10-CM U09.9) were excluded. We assessed the incidence of long COVID outcomes, based on diagnosis codes, during two periods – 31-197 days and 198-365 days after hospital admission – to determine if incidence of these outcomes changed over the year. Rates per 1000 person-years and 95% confidence intervals were calculated for all hospitalized patients, patients who had no record of those conditions in the year prior to admission, and immunocompromised patients. The percentage change in the rates for each symptom between the time periods was also calculated.

**Table 2.**
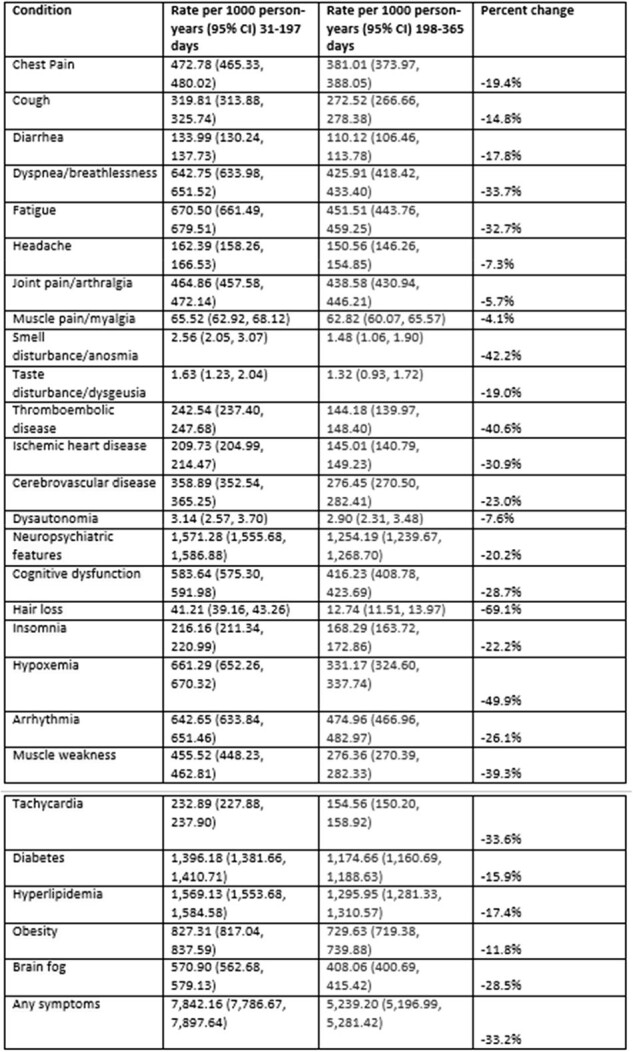
Rate of long COVID conditions per 1000 person-years in 31- to-197-day and 198- to- 365-day time periods post hospital admission, all hospitalized patients (N=92,913)

**Results:**

We identified 92,913 patients who met the study criteria. Cohort characteristics are described in Table 1; some outcomes were already present at baseline. In the overall hospitalized population (Table 2), incidence of all outcomes decreased between the first and second time periods. The decrease varied by symptom from 4% for muscle pain to 69% for hair loss. The trend was similar for the 12,100 patients who had none of the conditions at baseline (Table 3) and for the 39,185 immunocompromised patients (Table 4); some of the rare outcomes showed a nominal increase from the first to the second time periods, but this may be due to small sample sizes.

**Table 3.**
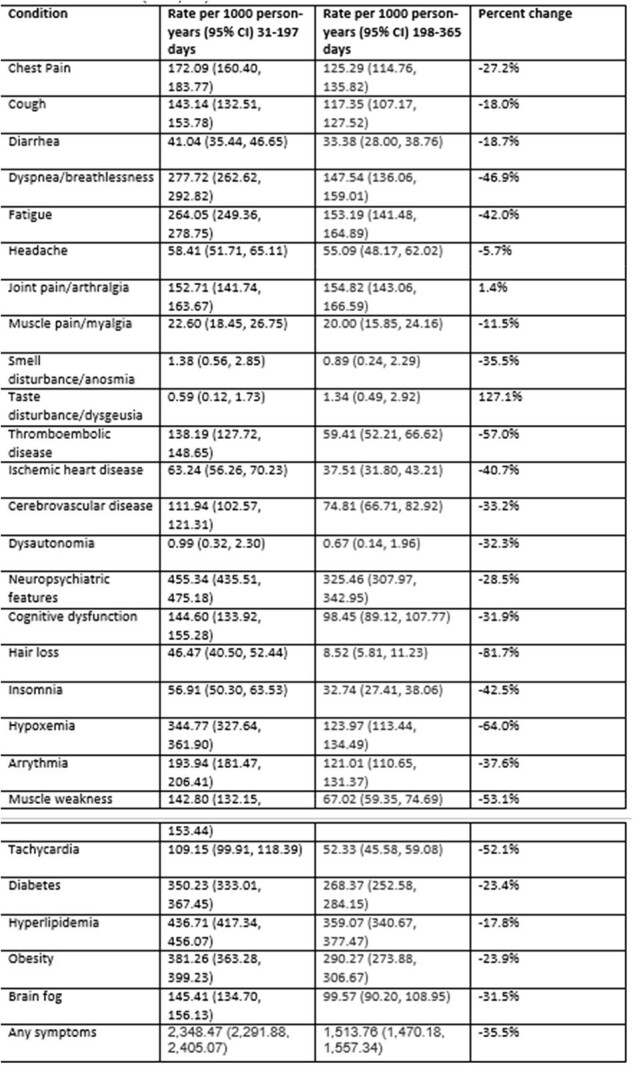
Rate of long COVID conditions per 1000 person-years in 31- to-197-day and 198-to- 365-day time periods post hospital admission, among hospitalized patients who had none of the conditions at baseline (N=12,100)

**Conclusion:**

The observed consistent tendency towards a decline in incidence over time indicates that many of these outcomes may have resulted from COVID-19 infection. Of note, incidence tended to decline more steeply for some conditions than others. This could be a result of an improvement in inflammatory response or decline in viral reservoir over the year. Additional research on drivers of long COVID over time is necessary.

**Table 4.**
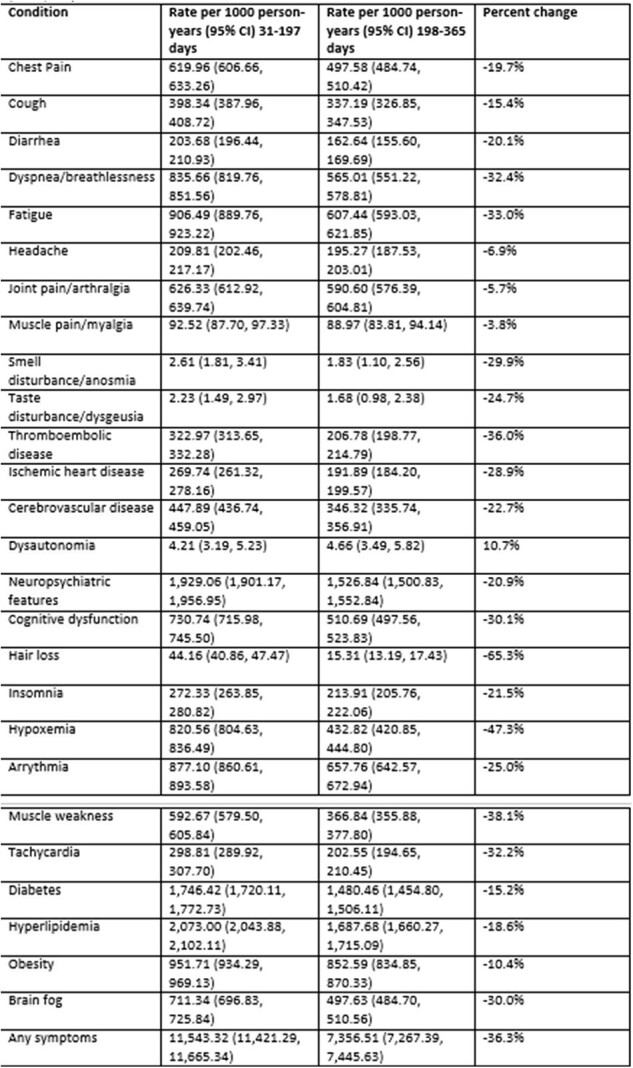
Rate of long COVID conditions per 1000 person-years in 31- to- 197-day and 198-to- 365-day time periods post hospital admission, among immunocompromised hospitalized patients (N=39,185)

**Disclosures:**

Mark Berry, PhD, Gilead Sciences, Inc.: Employee|Gilead Sciences, Inc.: Stocks/Bonds (Public Company) Valentina Shvachko, MS, Gilead Sciences: employee|Gilead Sciences: Stocks/Bonds (Public Company)|Gilead Sciences, Inc.: Employee of Gilead Sciences, Inc.|Gilead Sciences, Inc.: Stocks/Bonds (Public Company) Amanda Kong, DrPH, Aetion Inc: Employee|Aetion Inc: Stocks/Bonds (Public Company) Rohan Shah, BS, Aetion Inc: employee|Aetion Inc: Stocks/Bonds (Public Company) Gina Brown, MD, Gilead Sciences, Inc.: Employee of Gilead Sciences, Inc.|Gilead Sciences, Inc.: Stocks/Bonds (Public Company) Anand Chokkalingam, PhD, Gilead Sciences, Inc.: Employee|Gilead Sciences, Inc.: Stocks/Bonds (Public Company)

